# Does Rabbit Antithymocyte Globulin (Thymoglobuline®) Have a Role in Avoiding Delayed Graft Function in the Modern Era of Kidney Transplantation?

**DOI:** 10.1155/2018/4524837

**Published:** 2018-07-12

**Authors:** Lluís Guirado

**Affiliations:** Department of Renal Transplantation, Fundació Puigvert, Barcelona, Spain

## Abstract

Delayed graft function (DGF) increases the risk of graft loss by up to 40%, and recent developments in kidney donation have increased the risk of its occurrence. Lowering the risk of DGF, however, is challenging due to a complicated etiology in which ischemia-reperfusion injury (IRI) leads to acute tubular necrosis. Among various strategies explored, the choice of induction therapy is one consideration. Rabbit antithymocyte globulin (rATG [Thymoglobuline]) has complex immunomodulatory effects that are relevant to DGF. In addition to a rapid and profound T-cell depletion, rATG inhibits leukocyte migration and adhesion. Experimental studies of rATG have demonstrated attenuated IRI-related tissue damage in reperfused tissues, consistent with histological evidence from transplant recipients. Starting rATG intraoperatively instead of postoperatively can improve kidney graft function and reduce the incidence of DGF. rATG is effective in preventing acute rejection in kidney transplant recipients at high immunological risk, supporting delayed calcineurin inhibitor (CNI) introduction which protects the graft from early insults. A reduced rate of DGF has been reported with rATG (started intraoperatively) and delayed CNI therapy compared to IL-2RA induction with immediate CNI in patients at high immunological risk, but not in lower-risk patients. Overall, induction with rATG induction is the preferred choice for supporting delayed introduction of CNI therapy to avoid DGF in high-risk patients but shows no benefit versus IL-2RA in lower-risk individuals. Evidence is growing that intraoperative rATG ameliorates IRI, and it seems reasonable to routinely start rATG before reperfusion.

## 1. Introduction

 Delayed graft function (DGF) remains a major barrier to expanding the donor pool for kidney transplantation and improving outcomes. It is estimated to affect between 23% and 38% of deceased-donor adult kidney transplant recipients [1–3], based on the standard definition of dialysis during the first posttransplant week, and can increase risk of graft loss by up to 40% [4, 5]. The greatest impact on graft survival is seen in the first three months after transplant [6], but even beyond the first posttransplant year surviving grafts show impaired function [5] and there is a sustained increase in the risk of graft loss [4, 6]. Concerns about the risk of DGF restrict the acceptance of marginal grafts. A high proportion of kidneys recovered from donors aged 50 years or older, or from donors with high terminal creatinine, are discarded [7]. As patterns of donation change, for example, widening use of kidneys donated after circulatory death (DCD), and as the demographics of recipients and donors evolve, the question of how to avoid DGF becomes ever more pressing. Lowering the risk of DGF, however, is problematic due to its complicated etiology. In rare cases DGF may be caused by acute rejection [8], but far more commonly it arises from a complex interplay of events related to hypoxic and ischemic damage and reinstitution of blood flow after hypothermic preservation, with altered repair mechanism, that induce acute renal injury characterized by acute tubular necrosis (ATN) [8, 9]. Numerous risk factors have been identified (Table 1) [1, 3, 10–12], many of which are unmodifiable. Against this complex background, prevention is highly challenging [13]. Strategies focus on improving donor management and procurement techniques, new preservation methods such as pulsatile perfusion [14, 15], and tailoring of the immunosuppressive regimen to minimize early renal insults.

The choice of induction therapy is one consideration. Rabbit antithymocyte globulin (rATG) is generally used preferentially in patients at high immunological risk, such as sensitized individuals, and in patients with other risk factors for DGF including older donor age and longer cold ischemia time [16, 17]. As the profiles of recipients and donor change, and in the setting of modern preservation techniques and maintenance immunosuppressive regimens, do we know which kidney transplant should be considered most vulnerable to DGF and whether there is an adequate rationale for the choice of rATG? This review considers the available evidence.

## 2. Shifting Risk Profiles for DGF

Recent developments in the donor pool have affected the risk of DGF. Donor age, a known risk factor [3, 10], has remained relatively static but donor body mass index (BMI) is increasing [7]. In some countries, use of DCD donation has increased markedly, for example, from 7.3% in 2005 to 17.7% in 2015 in the USA [7]. DCD kidneys are more susceptible to ischemic injury, with a profound effect on risk of DGF. Irish* et al.* observed a threefold increase in DGF among recipients of a DCD graft in their analysis of Organ Procurement and Transplant (OPTN) data from 2003 to 2006 [1], while a UK study of controlled DCD transplants during 2001–2013 found the rate of DGF to be doubled (49% versus 25% with non-DCD donors) [18]. Expanded criteria donor (ECD) transplants, which by definition are from older donors, often with high terminal creatinine levels, are associated with a mildly elevated risk of DGF [19–21], although this effect has lessened in recent years, likely partly due to hypothermic machine perfusion [22]. For recipients, kidney allocation changes introduced in the US in 2014 have increased access to highly sensitized patients, leading to a significant 5% increase in rates of DGF [23].

The effect of these changes is illustrated by two studies which analyzed data from the OPTN database during different time periods, both published by the same group [1, 24]. The first included a cohort from 1995–1998 [24], while the second covered 2003–2006 [1], a period when the population was more highly sensitized, with an older mean donor age, and more transplants from DCD and ECD donors. The rate of DGF was 23.7% in the earlier cohort compared to 25.7% in the later study, reversing the previous decline in DGF observed during the 1990s [25].

## 3. Identifying Patients at Risk

*Clinical Assessment*. Scoring systems based on clinical features have been developed to determine which patients are most likely to develop DGF and have achieved a predictive accuracy of 70–75% [1, 12, 26]. These have all included recipient weight or BMI, donor age, and cold ischemia time, with or without recipient race, HLA mismatching, panel reactive antibody (PRA) status, donor terminal creatinine, DCD grafts, and type of induction therapy, and are convenient for use in routine practice.

*Donor Specific Antibodies (DSA)*. Preliminary evidence has pointed to a relationship between DGF and DSA. In a retrospective analysis of 771 kidney transplant patients at a single center, Peräsaari and colleagues found the incidence of DGF to be 48% in patients with pretransplant DSA versus 26% in nonsensitized individuals (p=0.0001), an association which remained significant on multivariate analysis (relative risk 2.04; p=0.005) [3]. Higher total pretransplant mean fluorescence intensity (MFI) values (3000–5000 MFI) increased risk versus levels of 1000–3000 [3].

*Donor Biomarkers*. High terminal serum creatinine is predictive of DGF [1, 12] but more accurate laboratory markers are emerging. Higher levels of neutrophil gelatinase-associated lipocalin (NGAL) and L-type fatty acid binding protein (L-FABP) in donor urine or perfusate [27, 28] can enhance prediction of DGF [27–29] and may become adopted in the future.

## 4. The Rationale for Use of rATG Induction

*Amelioration of Ischemia-Reperfusion Injury (IRI)*. IRI is the key process underlying the development of ATN. Beginning during the ischemic phase, with renal damage intensifying after reperfusion, it is characterized by epithelial and endothelial damage caused by tubular occlusion, impaired vascular flow, and various immunological and inflammatory responses [30, 31]. Attenuating IRI is clearly pivotal if DGF is to be avoided, and the immunological effects of rATG appear highly relevant [31]. rATG acts primarily by rapid and profound T-cell depletion induced by complement-dependent cell lysis, as well as by antibody-mediated cytotoxicity and activation-induced apoptosis [32], and downregulates cytokines that control T-cell activation [33]. These effects cause a dose-dependent depletion of CD2, CD3, CD4, CD8, CD20, and CD56 lymphocytes in the peripheral blood and secondary lymphoid tissues [33]. However, rATG also inhibits leukocyte migration [34] and downregulates leukocyte adhesion molecules [35, 36]. Primate models of IRI have shown that rATG reduces leukocyte adhesion in the endothelium and attenuates tissue damage in reperfused tissues [37, 38]. In a rat model of renal transplantation, anti-rat rATG given 2 hours prior to transplant prevented the tissue damage and tubular apoptosis associated with IRI and avoided early graft dysfunction [39]. Histology studies have shown that kidney transplant patients given rATG induction have less renal epithelial cell damage [40]. Recipients of a DCD liver graft exhibited less ischemic stricture formation in the biliary system when given rATG [34].

**Figure 1:**
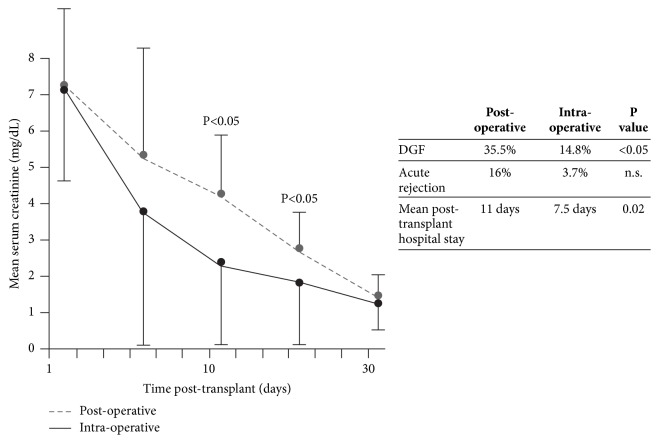
Serum creatinine to day 30 after transplant in kidney transplant recipients randomized to start rabbit antithymocyte globulin (rATG) intraoperatively (n=27) or postoperatively (approximately 6 hours after reperfusion, n=31) [41]. The total dose of rATG was 3–6 mg/kg in both groups. Maintenance immunosuppression comprised calcineurin inhibitor therapy (started based on renal function), mycophenolate mofetil (MMF), and steroids. DGF, delayed graft function.

**Figure 2:**
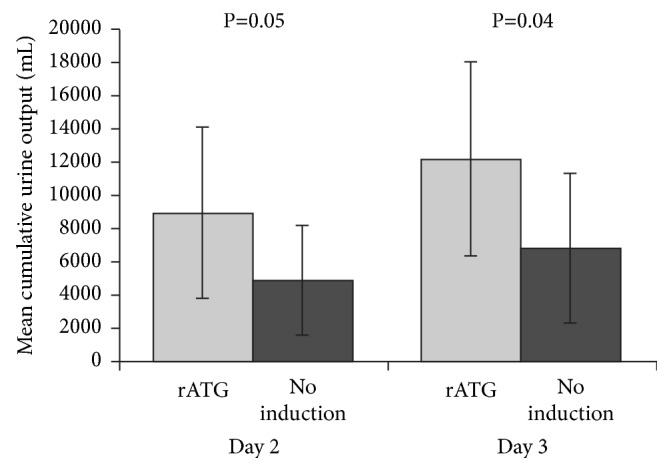
Cumulative urine output at day 2 and day 3 after kidney transplantation in patients receiving rabbit antithymocyte globulin (rATG) (n=7) versus no induction (n=11), with calcineurin inhibitor therapy initiated based on renal function, mycophenolate mofetil (MMF) and steroids [42]. Values are shown as mean (SD).

Clinically, two randomized studies in kidney transplantation have addressed the question of whether rATG can influence the impact of IRI on graft function. The first trial, by Goggins* et al.*, was performed to compare the incidence of DGF when rATG (total dose 3–6 mg/kg) was started intraoperatively or approximately six hours after reperfusion [41]. All 58 patients were given tacrolimus (initiated according to graft function), mycophenolate mofetil (MMF), and steroids. Risk factors for DGF were similar in both treatment arms. Early graft function was significantly better in the intraoperative cohort, DGF was less frequent, and the length of hospital stay was shorter versus the postoperative group (Figure 1). In 2005, McCune* et al.* performed a randomized trial of the vasodilator fenoldopam in 17 deceased-donor kidney transplant patients with cold ischemia time >12 hours [42]. They also analyzed early graft function in the 7 patients in the study given rATG (which was selectively administered to patients with peak PRA >40%, greater HLA mismatch and African-Americans) versus the 11 patients given no induction. rATG was started intraoperatively (1.5 mg/kg) with four subsequent daily doses of 1.5 mg/kg. Despite the unfavorable risk profile of the rATG-treated group, early urine output was significantly higher than in controls (Figure 2). In liver transplantation, a randomized single-center trial compared rATG (with the first dose of 1.5 mg/kg given intraoperatively) versus no induction and observed reduced IRI in the rATG arm, as indicated by significantly lower levels of liver enzymes on day 2 [43].

One final point of interest is an innovative study by Cicora* et al.* in a rat model of transplantation, where administration of anti-rat rATG to the donor prior to organ retrieval ameliorated IRI, as shown by lower necrosis and apoptosis scores and better early graft function [44]. This novel approach has not yet been examined by other researchers.

*Facilitating Delayed CNI Therapy.* A higher incidence of acute rejection in patients with DGF is well-documented [4, 45]. In a meta-analysis of 15 studies, Yarlagadda* et al.* found a relative risk of 1.38 for acute rejection in kidney transplant patients with DGF versus those without DGF [4]. Moreover, patients who experience both DGF and acute rejection have particularly poor outcomes, with fivefold increase in risk of graft loss by year 1 compared to patients without DGF [6]. Providing effective rejection prophylaxis is thus particularly important in patients at increased risk for DGF. Counterbalancing this, however, is the advantage of deferring the start of calcineurin inhibitor (CNI) therapy, typically for 4–5 days after transplant or until graft function has achieved a minimum threshold, in order to avoid CNI-induced vasoconstriction of the renal afferent arterioles [46] and CNI-related nephrotoxicity [47] as the graft recovers in the first few days after surgery.

The profound suppression of T-cells induced by rATG confers a potent immunosuppressive effect, and its efficacy in preventing acute rejection after kidney transplantation is well-accepted [48]. It is more effective than interleukin 2 receptor antagonist (IL-2RA) induction in patients at high immunological risk [49, 50], with similar efficacy in lower-risk individuals [51–54]. A recent analysis of the OPTN database showed that, overall, the risk for acute rejection by year 1 is higher with basiliximab than rATG (odds ratio 1.16; p<0.001) [55]. Additionally, rATG recipients showed longer survival and generally similar or better outcomes compared with alemtuzumab and basiliximab recipients [55]. rATG shows comparable efficacy to alemtuzumab in high-risk patients [56].

In 2001, Mourad* et al.* published a randomized trial in which 308 patients at varying levels of immunological risk received either rATG (at a total dose of 12.5 mg/kg, starting after surgery) with tacrolimus delayed until day 9 or no induction with tacrolimus started on the day of transplantation [57]. All patients received azathioprine and steroids [57]. Even with tacrolimus delayed for nine days, the incidence of biopsy-proven acute rejection (BPAR) was significantly lower in the rATG group (15.2% versus 30.4% in controls, p=0.001). However, the high rATG dose in this study, typical of dosing in the early 2000s, was associated with an unacceptable adverse events profile including more cytomegalovirus (CMV) infections, leukopenia, and thrombocytopenia [57]. Since then, five randomized trials have included a treatment arm using rATG induction with delayed CNI therapy [49, 51, 52, 54, 58]. All but one of these selected low to moderate immunological risk patients and demonstrated similar rates of BPAR and DGF in the rATG/delayed CNI groups versus IL-2RA induction with immediate [51] or delayed [52, 54] CNI therapy [49, 51, 52, 54]. In the other trial, rATG with CNI delayed to day 5 in both treatment arms was equally efficacious with or without oral steroids [58]. The only trial of rATG with delayed CNI undertaken in a high-risk population, by Brennan* et al.*, delayed cyclosporine (CsA) therapy until up to day 4 with either rATG or basiliximab induction [49]. Results showed a significantly lower incidence of BPAR (15.6% versus 25.5%, p=0.02) and steroid-resistant BPAR (1.4% versus 8.0%, p=0.005) under rATG induction (Table 2).

It seems reasonable to conclude that when CNI initiation is delayed in high-risk patients, rATG is preferable to IL-2RA induction, but that no advantage is offered for rATG versus IL-2RA induction in lower-risk individuals.

*Donor Specific Antibodies*. rATG preferentially inhibits reconstitution of pretransplant donor-reactive memory T-cells [59] and may induce apoptosis of plasma cells [60], the source of DSA. Patients with DGF have a higher incidence of* de novo* DSA (dnDSA) after transplant [61, 62] and it has been suggested that early dnDSA may contribute to intragraft injury, inhibiting graft recovery [63]. rATG is a component of many desensitization protocols and there is evidence to suggest that it also lowers the incidence of dnDSA after kidney transplantation [64]. Such an effect could be relevant to DGF, but more data are awaited.

## 5. rATG Induction and DGF: The Clinical Evidence

Few observational studies of risk factors for DGF have considered the effect of induction therapy, but Chapel* et al.* investigated a possible effect of rATG versus no rATG in a prospective study of 1,844 patients undergoing kidney transplantation in France after 2006 [12]. Exclusion criteria were living donation or a DCD graft, grafts preserved with pulsatile perfusion, preemptive transplantation, peritoneal dialysis pretransplant, and graft survival <7 days. The final model to predict DGF included five parameters, of which no rATG was one of the most important, with an odds ratio of 1.70 (Table 3). Within a subpopulation of 121 matched pairs with or without rATG induction, the odds ratio for DGF was similar to that seen in the overall population (odds ratio 1.66). Well-designed trials of rATG versus no induction are also rare. In the trial by Mourad* et al.*, in which 308 kidney transplant patients were randomized to rATG (12.5 mg/kg in total) with delayed tacrolimus or to no induction and immediate tacrolimus, the incidence of DGF was slightly lower in the rATG group (17.9% versus 24.1%) but the relevance of this protocol is limited.

Several randomized trials have compared outcomes with rATG versus IL-2RA induction in kidney transplant populations but interpretation can be complicated due to variations in population characteristics, dosing regimens and the starting time for CNI therapy (Table 2) [49–52, 54, 65, 66]. Two of these trials selectively studied patients at high immunological risk [52, 53]. In one of these, conducted by Noël* et al.*, there was a significant reduction in DGF under rATG induction versus daclizumab; importantly, rATG was stared intraoperatively, and tacrolimus was delayed in the rATG group but not the IL-2RA group [50]. Rates of BPAR and steroid-resistant BPAR were also significantly lower under rATG [50] (Table 2). When Brennan* et al.* compared rATG versus basiliximab in 278 patients selected for their high risk of acute rejection and DGF, rATG significantly lowered the rate and severity of BPAR compared to basiliximab (Table 2), but despite being started intraoperatively rATG did not affect the incidence of DGF [49]. Of note, CsA was delayed until up to day 4 in both treatment arms, and the authors also speculated that the long cold ischemia time (mean 26 hours) and advanced donor and recipient ages (mean 47 and 50 years, respectively) may have overwhelmed any protective effect. Other randomized trials have not selected high-risk patients for enrolment [51, 52, 54, 65, 66]—and indeed in many cases specifically excluded them [51, 52, 54, 65]—and in all but one case [65] did not start rATG until after surgery was completed. Rates of DGF were similar [52, 66] or lower [51, 54] with rATG induction, and effects on BPAR varied (Table 2). In the ongoing randomized PREDICT-DGF study, the primary endpoint is occurrence of DGF in patients randomized to rATG or basiliximab [67], and the results are awaited with interest.

Nonrandomized trials have also shown lower rates of DGF and BPAR compared to IL-2RA induction when rATG is started intraoperatively in patients at high immunological risk [68, 69], with mixed evidence for a benefit for DGF when rATG is initiated after surgery in mixed-risk populations [11, 70] or in low- or moderate-risk populations even if the first dose of rATG is given before reperfusion [71].

A randomized trial of alemtuzumab versus rATG in kidney-only or kidney-pancreas transplant recipients at low or moderate immunological risk, with induction started intraoperatively and with CNI therapy delayed in both groups, found no difference in rates of DGF and a lower incidence of BPAR under alemtuzumab [72]. Comparative studies in high-risk patients are lacking.

*DCD Transplants*. Mai* et al.* retrospectively compared outcomes in 40 DCD transplants versus 142 non-DCD kidney transplant recipients, unselected for immunological risk, who were all given rATG starting postoperatively (total dose 6 mg/kg) [73]. Maintenance therapy comprised immediate tacrolimus and MMF; steroids were withdrawn after day 5. Encouragingly, the incidences of both DGF and BPAR were similar in the DCD and non-DCD cohorts. Comparative studies of different induction agents in DCD transplants are scarce. Popat* et al.* prospectively collected data on 45 frequency-matched patients of any immunological risk level given rATG or IL-2RA induction [74]. rATG was started intraoperatively at a dose of 2.5 mg/kg, with one further dose of 1.25 mg/kg on day 4; both groups were given immediate CsA, MMF, and steroids. There was a trend to less frequent DGF and significantly fewer dialysis sessions, BPAR episodes, and readmissions in the rATG group (Table 4). Consistent with findings in non-DCD transplants, a retrospective study of 132 patients at a single center reported no difference in risk of DGF or BPAR with rATG versus IL-2RA induction in patients at low immunological risk when rATG was started after surgery [75].

Reviewing the results of these studies, rATG induction helps to prevent DGF when started intraoperatively with delayed CNI therapy in patients at high immunological risk, but not in other contexts.

## 6. rATG Dosing

In the last 15 years, rATG doses have declined from 10 to 12.5 mg/kg total dose used in the 1990s [76]. One comparative prospective analysis [77] demonstrated similar immunosuppressive efficacy using a total dose of 6 mg/kg to a dose of 10.5 mg/kg [78]. A cumulative dose of 6 mg/kg is now typical, with lower doses (3–6 mg/kg) often used in low-risk patients [76]. There is a lower limit, however: one pilot study in a cohort of 45 low-risk patients found a higher rate of DGF (40% versus 14.3%, p=0.05) when the total dose of rATG was reduced to 2.25 mg/kg versus 3.75 mg/kg, both started intraoperatively [79]. In high-risk patients, it would seem prudent to give a cumulative dose of 6 mg/kg [76]. At our center, we give a dose of 1.25 mg/kg (recently started intraoperatively), with subsequent doses adjusted according to daily lymphocyte counts: it is increased by 25 mg/kg if the count is >250/mm^3^, maintained at 1.25 mg/kg if the count is 100–250/mm^3^, and reduced by 25 mg/kg if the count is <100 mm^3^. The maximum total dose is 6 mg/kg (rATG is not continued beyond day 7) and tacrolimus is initiated on day 5, or earlier if serum creatinine reaches 300 *μ*mol/L. Alternatively, the rATG dose can be adjusted to maintain CD2/CD3 count in the range 10–20/mm^3^ or continued until serum creatinine declines to an acceptable level (e.g., <300 *μ*mol/L).

Experience from liver transplantation suggests that any single intraoperative dose of rATG should by less than 3 mg/kg [80]: 1.5 mg/kg is almost universally used when given during surgery.

## 7. Conclusions

Avoiding DGF is a key clinical objective in kidney transplantation. As recipient and donor demographics change—and new policies such as use of DCD grafts are introduced—the need to harness all possible strategies to reduce DGF becomes ever more pressing. The multifaceted immunomodulatory effects of rATG induction mean it is the preferred choice for supporting delayed introduction of CNI therapy in high-risk patients, and evidence is growing that intraoperative rATG ameliorates IRI, the pathological basis for ATN and DGF. The ongoing PREDICT-DGF study [67] will provide further data.

The potential benefits of rATG must, of course, be balanced against the risk of side effects, conventionally related to concerns about rates of infection or malignancy (particularly posttransplant lymphoproliferative disease [PTLD]). The higher doses of rATG which were used in the 1990s and early 2000s and which were associated with increased complications [81] have since been progressively lowered [76]. Well-designed studies in which the total rATG dose was ≤7.5 mg/kg [49, 65, 74] have not indicated any increase in the incidence of infections versus IL-2RA induction other than a higher overall incidence of infection but a lower rate of CMV infection under rATG in the study by Brennan et al. [49]. Recent reviews and registry analyses have concluded that with contemporary dosing regimens (total dose ≤6 mg/kg) rATG induction is not associated with an enhanced risk for PTLD or cancer [82–84]. In terms of expenditure, a recent analysis concluded that rATG or basiliximab induction incurs similar treatment costs over the first two years after kidney transplantation based on data from the high-risk population included in the randomized trial by Brennan* et al.* [85]. Analyses have not been reported in lower-risk cohorts.

Defining the contribution of a single intervention to the risk of DGF is difficult given the wide variations in donor management, graft quality, patients' risk status, donor quality, and immunosuppressive protocols. The current evidence base does not indicate a significant benefit for rATG induction in terms of preventing DGF in lower-risk patients; here, IL-2RA induction seems to be equally effective. However, there are now sufficient data to conclude that rATG induction—started intraoperatively—can help to avoid DGF in high-risk patients, reinforcing its benefit in the lowering of BPAR in these vulnerable individuals.

## Figures and Tables

**Table 1:** Key risk factors for delayed graft function [1, 3, 10–12].

**Donor characteristics**	**Recipient characteristics**	**Immunological factors**

Older age	Female gender	ABO incompatibility

Higher body mass index	Higher body mass index	Higher HLA mismatching

Higher terminal creatinine	African-American race	Higher panel reactive antibody levels

Donation after cardiac death	Diabetes	Previous transplant
	Dialysis at time of transplant	Pretransplant DSA

DSA, donor specific antibodies.

**Table 2:** Randomized clinical trials of rATG versus IL-2RA induction in adult kidney transplant recipients. Induction and maintenance therapies were started after procedure on the day of transplantation unless otherwise stated.

Study	Population	n	rATG regimen	IL-2RA regimen	DGF (rATG vs IL-2RA)	BPAR (rATG vs IL-2RA)	Other findings (rATG vs IL-2RA)
Thomusch et al., 2016 [65]	Low immunological risk (including no pretransplant DSA, PRA ≤30%)	615^*∗*^	rATG (1.5 mg/kg intraoperatively, 1.5 mg/kg x 3 to day 3) Immediate low- dose TAC MMF Steroids to day 8	Basiliximab (20 mg/kg intraoperatively, 20 mg day 4) Immediate low- dose TAC MMF Steroids to day 8	Not stated	9.9% vs 10.6% (p=0.87)	-

Pilch et al., 2014 [66]	Unselected (other than ABO compatible)	200	rATG (5 x 1.5 mg/kg) Immediate TAC MMF Steroids	Daclizumab (2 x 1 mg/kg)^*∗∗*^ Immediate TAC MMF Steroids	9% vs 10% (p=0.81)	Month 6: 2% versus 8% (p=0.05)	Mean time to BPAR by month 12: 98 vs 241 days (p<0.001)

Noël et al., 2009 [50]	High immunological risk (including PRA ≥30% or peak PRA ≥50%), no positive T-cell cross-match or DCD	227	rATG (1.25 mg intraoperatively and on days 1- 7) TAC from day 2-5 MMF Steroids	Daclizumab (5 x 1 mg/kg) Immediate TAC MMF Steroids	31.5% vs 44.6% (p=0.044)	Month 12: 15.5% vs 27.2% (p=0.016)	Steroid-resistant BPAR at month 12: 2.7% vs 14.9% (p=0.002)

Abou-Ayache et al., 2008 [54]	Low or moderate immunological risk (including CIT ≤36 hours, PRA ≤20%), deceased donor	113	rATG (dose adjusted based on CD2/CD3 count) CsA started by day 7 (or earlier depending on graft function) MMF Steroids	Daclizumab (2 mg/kg pretransplant, 1 mg/kg on day 14) CsA started by day 7 (or earlier depending on graft function) MMF Steroids	12.7% vs 18.5%^†^	Month 12 14.5% vs 16.7% (n.s.)	Mean time to BPAR by month 12: 82 vs 133 days (n.s.)

Brennan et al., 2006 [49]	High risk for acute rejection or BPAR^‡^	278	rATG (1.5 mg/kg intraoperatively, then 1.5 mg/kg x 4) CsA started by day 4 (or earlier depending on graft function) MMF Steroids	Basiliximab (2 x 20 mg) CsA started by day 4 (or earlier depending on graft function) MMF Steroids	40.4% vs 44.5% (p=0.54)	Month 12: 15.6% vs 25.5% (p=0.02)	Steroid-resistant BPAR at month 12: 1.4% vs 8.0% (p=0.005)

Mourad et al., 2004 [52]	Low or moderate immunological risk (including PRA ≤20%), deceased donor	105	rATG (1 mg/kg on days 0 and 1, then based on CD3 count) CsA according to renal function MMF Steroids	Basiliximab (2 x 20mg) CsA according to renal function MMF Steroids	30.2% vs 28.8%	Month 12: 9.4% vs 9.6% (n.s.)	Significantly more frequent CMV infections, leukopenia and thrombocytopenia with rATG vs basiliximab

Lebranchu et al., 2002 [51]	Low or moderate immunological risk (including CIT ≤36 hours, PRA ≤25%, no T-cell cross-match)	100	rATG (dose adjusted based on CD2/CD3 count) CsA (started based on graft function) MMF Steroids	Basiliximab (2 x 20mg) Immediate CsA MMF Steroids	6% vs 14%^†^	Month 12: 8.0% vs 8.5% (n.s.)	Significantly more frequent CMV infections with rATG vs basiliximab

^*∗*^A third treatment group comprised basiliximab with low-dose TAC, MMF, and steroid maintenance therapy (data not shown).

^*∗∗*^No p value provided.

^†^Amended to basiliximab (2 x 20 mg) after withdrawal of daclizumab from the market.

^‡^Based on duration of cold ischemia time and predefined donor/recipient risk factors.

BPAR, biopsy-proven acute rejection; CIT, cold ischemia time; CMV, cytomegalovirus; CsA, cyclosporine; DCD, donation after circulatory death; DGF, delayed graft function; DSA, donor-specific antibodies; IL-2RA, interleukin-2 receptor antagonist; MMF, mycophenolate mofetil; n.s., not significant; PRA, panel reactive antibodies; rATG, rabbit antithymocyte globulin; TAC, tacrolimus.

**Table 3:** Variables included in a predictive model for DGF based on 1,844 deceased-donor transplants since 2007 [12].

**Variable**	**Odds ratio**	**95**% **CI**	**P value**

Cold ischemia time (hours)	1.06	1.04, 1.08	<0.0001

Donor age (years)	1.02	1.01, 1.02	0.0014

BMI (kg/m^2^)	1.06	1.02, 1.09	0.0004

Donor creatinine >108 *µ*mol/L	1.76	1.29, 2.41	0.0004

No rATG	1.70	1.30, 2.23	0.001

BMI, body mass index; CI, confidence interval; DGF, delayed graft function; rATG, rabbit antithymocyte globulin.

**Table 4:** Results of a retrospective analysis of prospectively collected from 45 frequency-matched DCD kidney transplant patients given rATG (2.5 mg/kg intraoperatively and 1.25 mg/kg on day 4) or daclizumab (2 x 1 mg/kg) as induction, with immediate CsA, MMF and steroids [74].

	**rATG** **(n=24)**	**Daclizumab** **(n=21)**	**P value**

DGF, %	52	65	0.08

Dialysis sessions, n	38	62	0.0001

Hospitalized days post-transplant, n	95,600	167,200	0.0004

BPAR, %	0	13	0.003

Mean healthcare cost per patient by year 1 (£)	14,904	18,929	0.002

BPAR, biopsy-proven acute rejection; CsA, cyclosporine; DCD, donation after cardiac death; MMF, mycophenolate mofetil; rATG, rabbit antithymocyte globulin; DGF, delayed graft function.

## Abbreviations

ATN: Acute tubular necrosis
BMI: Body mass index
BPAR: Biopsy-proven acute rejection
CMV: Cytomegalovirus
CNI: Calcineurin inhibitor
CsA: Cyclosporine
DCD: Donation after circulatory death
DGF: Delayed graft function
dnDSA: De novo donor specific antibodies
DSA: Donor specific antibodies
ECD: Expanded criteria donor
IRI: Ischemia-reperfusion Injury
IL-2RA: Interleukin-2 receptor antagonist
L-FABP: L-type fatty acid binding protein
MFI: Mean fluorescence intensity
MMF: Mycophenolate mofetil
NAGL: Neutrophil gelatinase-associated lipocalin
OPTN: Organ Procurement and Transplant
PRA: Panel reactive antibody
PTLD: Posttransplant lymphoproliferative disease
rATG: Rabbit antithymocyte globulin (Thymoglobuline).
